# Patterns and Determinants of Weight Gain among People Who Use Drugs Undergoing Treatment for Recovery in Lebanon

**DOI:** 10.3390/nu15040990

**Published:** 2023-02-16

**Authors:** Nadine Mahboub, Rana Rizk, Cynthia George Farsoun, Nanne de Vries

**Affiliations:** 1 Department of Nutrition and Food Sciences, Lebanese International University, Beirut P.O. Box 146404, Lebanon; 2 Department of Health Promotion, CAPHRI School for Public Health and Primary Care, Maastricht University, P.O. Box 616, 6200 Maastricht, The Netherlands; 3 Department of Natural Sciences, School of Arts and Sciences, Lebanese American University, Byblos P.O. Box 36, Lebanon; 4 Institut National de Santé Publique, d’Epidémiologie Clinique et de Toxicologie-Liban (INSPECT-LB), Beirut, Lebanon; 5 Department of Nursing and Health Sciences, Notre Dame University, Zouk Mosbeh P.O. Box 72, Lebanon

**Keywords:** drug use disorder, drug abuse treatment centers, determinants of weight gain, healthy lifestyle, nutritional status, Lebanon

## Abstract

Substance use disorder compromises the nutritional status and the eating habits of drug users, often leading to malnutrition. Once referred for treatment, hyperphagia and poor lifestyle practices leading to weight gain are observed. This study aimed to examine the patterns and extent of weight change as well as the determinants of weight gain in a sample of drug users who were receiving treatment in Lebanon. A total of 172 male participants undergoing either rehabilitation or opioid substitution treatment (OST) were included. Multivariate regression analysis was applied to assess the effect of different variables on weight gain while adjusting for potentially confounding variables. Approximately two-thirds (65.1%) of the participants gained weight (OST: 54.3%, rehabilitation: 78.2%; *p* < 0.05). The mean weight gain was 5.9 kg and was mainly reported among participants in the underweight, normal, and overweight pre-treatment categories and accentuated in the rehabilitation group (OST: 2 kg, Rehabilitation: 10.6 kg). Around half of the participants moved from the normal weight category to the overweight and obese categories during treatment. Weight gain was negatively associated with the number of previous treatment attempts (Odds Ratio = 0.86; Confidence Interval: 0.74–0.99), duration of current treatment (Odds Ratio = 0.98; Confidence Interval: 0.96–0.99), and pre-treatment body mass index (BMI) (Odds Ratio = 0.88; Confidence Interval: 0.80–0.96). Investigating other nutrition and lifestyle practices, neither nutrition knowledge, food addiction, physical activity level, nor sleep quality were associated with weight gain. Treatment through drug use was associated with meaningful weight gain that might lead to health risk factors. Developing health promotion programs is crucial to enhance treatment and decrease the risk of relapse.

## 1. Introduction

Substance use disorder (SUD) has long been known to impair the nutritional status and dietary habits of people who use drugs (PWUD) [1,2]. The majority of the literature generally points towards undernutrition [2,3,4] associated with decreased intake, poor dietary behaviors, [5,6] and anthropometrics below standard values [2,7,8,9,10].

Worldwide, more than a quarter of a billion people are estimated to suffer from SUD, requiring treatment [11]. The main treatment modalities providing evidence-based behavioral and pharmaceutical therapy include rehabilitation (detoxification or complete abstinence) and opioid substitution treatment (OST) (medication-assisted program). In-patient residential rehabilitation follows a detoxification program in a hospital. The centers follow strict, disciplined daily schedules for eating, sleeping, and performing chores. On the other hand, OST is organized in outpatient centers that provide PWUD with a pharmaceutical opioid agonist (buprenorphine or methadone).

After the initiation of treatment, whether rehabilitation or OST, lifestyle and nutritional behaviors start to change. Hyperphagia and binging on sugars and fats as substitutes for drugs, low physical activity, and inadequate sleep quality and quantity and food addiction are observed in both treatment modalities [3,12,13,14,15,16]. The disruption of sleep during periods of drug withdrawal can be attributed to several factors such as psychopathological problems, smoking, and the duration of drug use [17]. Another lifestyle factor that is impacted by drug use is physical activity. The scarce studies are controversial; some showed the involvement of PWUD in structured physical activity regimens, while others showed little participation [18]. Nevertheless, physical activity remains an important element in the treatment from substance use as it decreases withdrawal symptoms, depression, and increases self-confidence [19]. Food addiction has not been largely studied among PWUD undergoing treatment for recovery. The dearth of studies conducted on patients undergoing methadone maintenance treatment (MMT) show a relation between low food addiction and higher nutrition knowledge [16]. Subsequently, a trend of weight gain is detected, that might be attributed to binge eating [20], compromised neurological mechanisms in the brain causing food addiction [21,22,23,24,25], poor sleep, and medications used to assist in recovering from SUD [3,26,27,28]. Weight gain during MMT (MMT) may be directly attributed to methadone itself [9]. Exposure in opiate agonists is associated with a shift in dietary preferences towards sweet and fatty foods [29], as well as glycemic control derangement [30].

Higher risks of chronic diseases such as diabetes, cardiovascular diseases, and psychological disorders in healthy individuals have been linked to weight gain and unhealthy lifestyle choices [31,32,33]. The association between weight gain and non-communicable diseases among people with SUD has been scarcely studied. Sweeney et al. [34] found that the percentage of patients with cardiovascular risk factors increased with increasing body mass index (BMI) during MMT. Additionally, a possible trigger for relapse, especially in females, is body dissatisfaction brought on by weight gain. [3,35,36,37].

Although the majority of the literature points towards weight gain during treatment for SUD [12,36,38], little is known about this population group’s weight change trends and magnitude. Some recovering patients experience further weight loss, while others experience no weight change [39]. In addition, significant differences concerning weight gain have been observed across different treatment modalities [39]. Determinants of weight gain during treatment remain understudied. However, such data are crucial to inform health promotion and weight gain prevention measures during treatment, ultimately improving treatment outcomes and preventing relapse.

Lebanon is a small country in the Eastern Mediterranean region with a high middle- class income. Internal and regional armed conflicts were motivating factors for an extensive drug use that was higher than the global average [40]. Furthermore, Lebanon serves as a transit country for the trafficking of drugs in combination with a high local production [41]. All of these factors lead to the wide availability of illicit drugs and a lack of control over their consumption [42].

This study aimed to investigate the patterns and extent of weight change in PWUD undergoing treatment for recovery in Lebanon and to analyze how these variables differ between those receiving OST and those receiving rehabilitation. Furthermore, we also aimed to investigate the determinants of weight gain in this sample.

## 2. Methods

This cross-sectional study was carried out in rehabilitation and opioid substitution drug treatment centers in Lebanon. The details of the study are presented in Mahboub et al. [39]. Four OST centers and seven rehabilitation centers were approached. A convenience sample was chosen, as only three OST centers and four rehabilitation centers agreed to participate. Participants included Lebanese adults over the age of 18 years, who had been undergoing treatment for more than a month. A total of 187 participants met the inclusion criteria and participated in the original study. Due to the small female sample size, only males were included in this study (OST: *n* = 94; rehabilitation: *n* = 78).

All PWUD treated in these centers were informed of the study’s goals and methodology, as well as their right to withdraw at any time. Ethical approval was obtained by the Lebanese International University’s Committee on Research Ethics (CRE) (case number: LIUIRB-180122-NB1). Written informed consent was obtained from all participants.

### 2.1. Data Collection

Data collection was conducted in the treatment centers by trained licensed dietitians between January 2018 and March 2019 and required 40–50 min per participant.

### 2.2. Collected Data Included

Demographic information, medical history, and history of drug use were investigated. The questionnaire was based on information from the literature about the dietary habits, nutritional state, and lifestyle of PWUD patients or those undergoing treatment for recovery.

Anthropometric measurements: trained, licensed dietitians assessed the participants for anthropometrics. (1) Height (cm) measured to the nearest 0.1 cm without shoes using a portable digital wall-mounted height scale; (2) Weight (kg) measured without shoes and wearing just light clothing using a calibrated mechanical floor scale. BMI was computed as the ratio of weight (kg) to height squared (m^2^). The BMI classification was as follows: underweight (<18.5 kg/m^2^), normal weight (18.5–24.9 kg/m^2^), overweight (25–29.9 kg/m^2^), and obese (>30 kg/m^2^) (WHO, 2000).

The difference between the self-reported usual pre-treatment body weight (kg) and the measured bodyweight (kg) on the assessment day was used to calculate the self-reported weight change (kg).

Dietary intake measures were evaluated using the Multiple Pass Food Recall (MPR), a 24 h food recall system developed by the United States Department of Agriculture that lessens the recall bias [43,44]. The Nutritionist Pro software’s food composition database (Nutritionist Pro, Axxya Systems, San Bruno, CA, USA, version 5.1.0, 2018) was used to calculate the daily consumption of energy, macronutrients, and micronutrients of the participants’ 24 h recollections. The database’s software was enhanced with the addition of regional food recipes [45]. The values from the data that was analyzed were compared to the US-based Dietary Reference Intakes (DRIs), as recommended by the Institute of Medicine (Dietary Reference Intake Tables), due to the absence of gender- or age-specific DRIs for the Middle Eastern populations.

Nutrition knowledge: measured using the Consumer-Oriented Nutrition Knowledge Questionnaire (CoNKQ), which was adapted from Spillman and Keller [46]. This is a validated questionnaire, with good internal reliability (Cronbach’s alpha: 0.743), and construct validity. It comprised 20 detailed questions that were compiled from surveys and professional advice on healthy eating. A score of less than 60% was defined as poor knowledge.

Food addiction: The Yale Food Addiction Scale (YFAS) was used to assess food addiction among the participants [47]. This highly reliable scale (Cronbach’s alpha: 0.84) was developed to detect food addiction to certain types of foods (high fat and high sugar) among individuals. The past year’s eating habits were reviewed, along with the definitions of food addiction that were translated in relation to eating behaviors (such as withdrawal symptoms, difficulties cutting down, tolerance, etc.). Food addiction is diagnosed when three or more of the mentioned eating behavior symptoms were present within the past 12 months.

Sleep quality was assessed using the Pittsburgh Sleep Quality Index (PSQI) [48]. The questionnaire consisted of four questions to estimate the duration of sleep, the time needed to fall asleep, the time needed to wake up, in addition to the duration spent in bed immediately after waking up. Moreover, five other questions were used to identify reasons for troubled sleep. Answers to these questions were converted into a total score, where a score of 5 or more indicated poor sleep quality and a score between 0 and 4.9 indicated good sleep quality.

Physical activity: The International Physical Activity Questionnaire (IPAQ) was used to assess the physical activity of the participants. The duration and frequency of physical activity (light, moderate, and vigorous) in the past seven days were measured by seven questions [49].

The Arabic version of the PSQI, that was culturally adapted by Haidar et al., was used [50]. The other questionnaires used in this study were translated to Arabic and followed the recommended process [51].

### 2.3. Statistical Analysis

Data analysis was conducted using the Statistical Package for the Social Sciences (SPSS) version 24.0. Means and standard deviation (SD) were used to report continuous data, and frequencies (N) and percentages were used to report categorical data. The Kolmogorov–Smirnov test was used to test for the normality of the data. For continuous variables with a normal distribution, the independent samples t-test was used to compare data, while the Mann–Whitney U test was used for variables with a skewed distribution. A chi-square test was used to compare data for categorical variables. Multivariate regression analysis was applied to assess the effect of different variables on weight gain while adjusting for potentially confounding variables. First, a bivariate analysis was conducted to explore sociodemographic, drug use, treatment-related, nutrition, and lifestyle variables associated with weight gain (reference category: no weight gain). Variables with *p*-values less than 0.2 were kept for the regression analysis. Second, a stepwise regression analysis assessed the association between weight gain (yes versus no) and the different determinants. Variables included in the model were: number of previous treatment attempts; duration of current treatment in months; type of treatment (reference: OST); current use of any medication (reference: no); pre-treatment BMI in kg/m^2^; food addiction (reference: no diagnosis); nutrition knowledge (reference: good knowledge); sleep quality (reference: good sleep quality); physical activity (reference: high). Statistical significance was indicated by a *p*-value < 0.05. Odds ratios and 95% confidence intervals were calculated.

## 3. Results

Basic demographic information and the medical and drug use history of the sample are presented in Table 1. The mean age of the population was 33 years. A total of 30% of the participants had a secondary or university level of education, with 44.2% being unemployed at the time of data collection. The use of nervous system drugs was common among the participants. Around half of the participants (52.6%) in rehabilitation treatment had never previously been admitted to recovery, compared with 37.2% in the OST group (*p* < 0.05). Furthermore, more than three-quarters of the population (77.3%) was only addicted to drugs, while 15.1% had alcohol drinking problems, with this problem being more prevalent in the rehabilitation group (*p* < 0.05). On average, the participants had previously attempted treatment nearly three times and were on treatment for around 25 months. The treatment duration was significantly higher in the OST group.

Table 2 details the anthropometric and lifestyle practices of the participants. The average BMI of the participants increased from 25.9 ± 5.2 kg/m^2^ to 27.4 ± 5.5 kg/m^2^. The mean energy intake of the sample was 2641.9 kcal/day, with no statistical difference across treatment modalities. Around two-thirds of the participants (65.1%) reportedly gained weight; this finding was more common in the rehabilitation group (OST: 54.3%, rehabilitation: 78.2%; *p* < 0.05). Three-quarters (75.4%) of the participants had poor sleep quality, this result was more frequent in the rehabilitation group. Moreover, 50.6 percent of participants engaged in little physical activity. This was more common in the OST group. Finally, there was a food addiction diagnosis for half the participants and the majority had poor nutrition knowledge (49.1% and 68.6%, respectively).

The total reported weight change, weight change per pre-treatment BMI category, and weight change per treatment method are detailed in Table 3. The mean weight gain of the participants was 5.9 ± 12.4 kg (OST: 2.0 ± 11.3 kg; rehabilitation: 10.6 ± 12.0 kg). Participants who were underweight, normal weight, or overweight prior to treatment all gained weight. In contrast, participants who were obese before treatment in the OST group lost weight during treatment, while those in the rehabilitation group reportedly gained weight.

**Figure 1:**
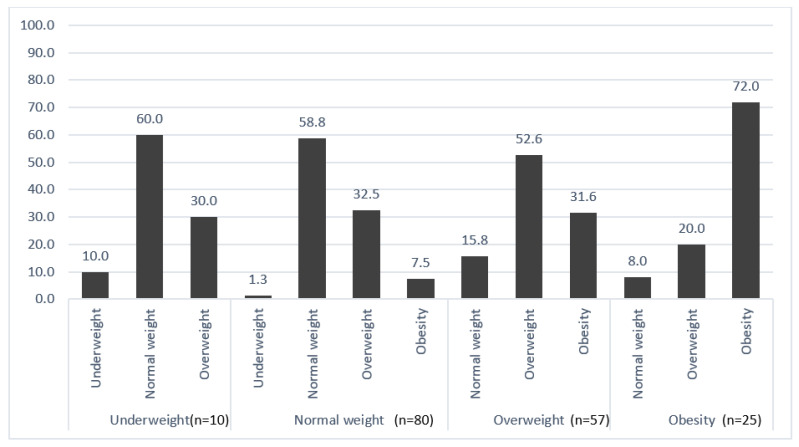
BMI changes pre- and during treatment (*n* = 172). Pre-treatment BMI (Horizontal), during treatment BMI (vertical). Values are presented as percentages.

Figure 1 illustrates the trends of weight change during treatment across BMI categories in the total sample. Out of 10 participants in the pre-treatment underweight category, six (60%) normalized their weight, whereas three (30%) became overweight. Furthermore, 32 participants out of 80 (40%) who had normal pre-treatment weight moved to the overweight and obese categories, whereas 31.6% of the overweight participants became obese. On the contrary, around 28% of the initially obese participants moved to the overweight and normal BMI categories during treatment.

**Figure 2:**
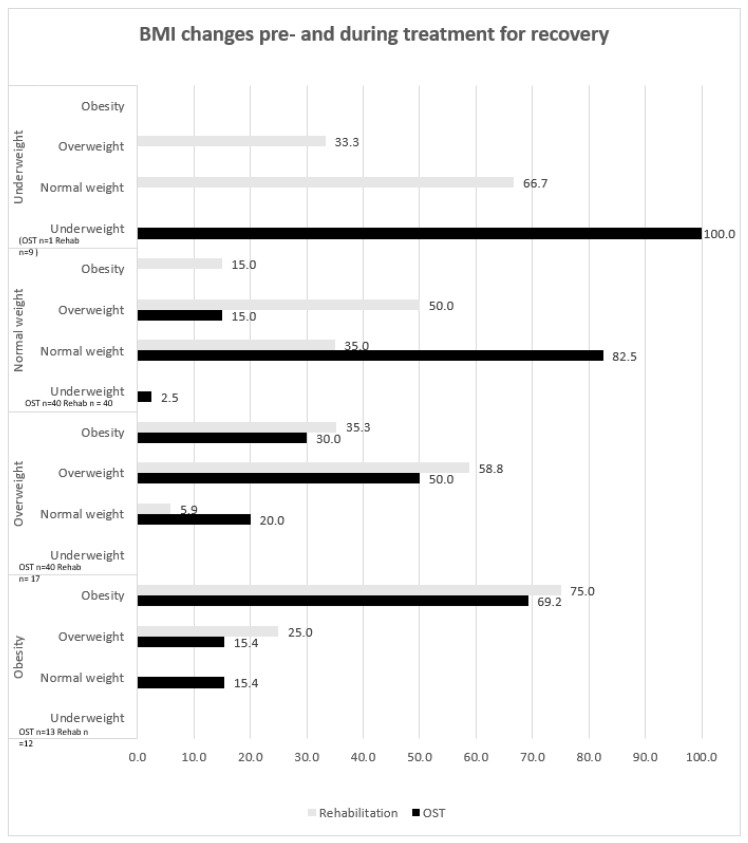
BMI changes pre- and during rehabilitation (*n* = 78) and OST (*n* = 94). Pre-treatment BMI (vertical), during treatment BMI (horizontal)**.** Values are presented as percentages.

The percentages of participants changing their BMI category during -treatment in each rehabilitation and OST centers are illustrated in Figure 2. The weight gain trend was more accentuated in the rehabilitation group compared with the OST group. Three participants out of nine in the underweight category became overweight (33.3%). Moreover, the majority of the participants, 26 out of 40 (65%) within the normal weight BMI category in the rehabilitation group, moved to the overweight and obese category during treatment, as opposed to six out of 40 participants (15%) in the OST group. Furthermore, 35.3% and 30% of the participants in the overweight BMI category from the rehabilitation and OST groups, respectively, became obese. Finally, 30.8% and 25% of the obese OST and rehabilitation participants, respectively. moved to the overweight and normal weight BMI categories.

The bivariate associations between sociodemographic, drug use and treatment method, nutrition and lifestyle practices, and weight gain are illustrated in Table 4. The number of treatment attempts and duration of current treatment were significantly lower among participants who reported weight gain. Furthermore, more participants reporting weight gain were in rehabilitation treatment compared with OST and were currently using medications. Finally, pre-treatment BMI was significantly associated with weight gain in the studied sample. No significant associations were observed with other lifestyle practices.

Determinants of weight gain are detailed in Table 5. Weight gain was negatively associated with the number of previous treatment attempts (OR = 0.86; CI: 0.74–0.99), the duration of current treatment (OR = 0.98; CI: 0.96–0.99), and the pre-treatment BMI (OR = 0.86; CI: 0.79–0.95).

## 4. Discussion

The Middle East and North Africa (MENA) region hosts the world’s largest manufacturer of opioids, in addition to containing the main drug trade routes [52]. Subsequently, the region faces a large drug use problem due to the increasing accessibility of cheap heroin [53]. In Lebanon, a part of the MENA region, drug abuse has progressed in the country following the civil war due to the easy access to drugs, socioeconomic instabilities, and the vulnerability of the population after the armed conflicts [40]. At the same time, Lebanon hosts World Health Organization (WHO)-designed knowledge hubs related to PWUD for the region [41]. Studies examining the dietary habits, nutritional status, and lifestyle practices of PWUD undergoing treatment for recovery in the MENA region, specifically Lebanon, are scarce. Mahboub et al. [39] reported excessive weight gain in addition to inadequate knowledge, high levels of food addiction, poor sleep quality, and low levels of physical activity among PWUD in different treatment centers in Lebanon. To our knowledge, the patterns of weight change and studying the determinants of weight gain in PWUD undergoing different treatment modalities are not addressed in the literature. Such studies are of great importance for the development of health intervention programs aiming at improving health status and preventing relapse among this population group.

As a result, this paper is a pioneer in researching weight change patterns and determinants of weight gain among PWUD in Lebanon treatment centers. Our sample consisted of 172 male participants undergoing either rehabilitation or OST. The main findings reflect that weight gain was significantly higher in the rehabilitation group, among people with fewer treatment attempts and people who were either underweight, normal, or overweight pre-treatment. Further studies in Lebanon and in the region are warranted for the comparability and generalizability of the results among all PWUD undergoing treatment for recovery.

Our results in general show high energy intake and weight gain among the participants in both treatment modalities. This finding is supported by many studies showing weight gain and an increase in BMI among PWUD undergoing methadone maintenance treatment (MMT) [36,54,55] and residential rehabilitation [13,56]. This increase in weight may be related to engaging in atypical eating habits, where patients substitute their obsession with drugs with that of sweet taste, seeking common euphoric rewards [3,57], impulsive unhealthy eating patterns following drug cessation leading to increased energy intake [58], and the impact of the psychotropic medications prescribed [3,30,59]. Several studies have also reported that MMT practitioners encounter greater consumption of sweet and other palatable foods, leading to higher energy intakes and thus, weight gain and higher BMIs [24,53]. Sugar consumptions has been shown to increase endogenous opiates, an underlying motivational reward mechanism in the brain [60]. This suggests that there may be treatment-related factors influencing BMI changes and weight gain [53]. The link between drugs and food merits further investigation, as it is still unclear whether taste preferences are due to drug use or vice versa [24]. Moreover, weight was regained among patients who attempted to lose weight regardless of the dietary and behavioral modification interventions used [61]. This is attributed to several mechanisms that the body uses to oppose the weight loss. The decrease in energy expenditure during weight loss, known as adaptive thermogenesis, increases hunger thus promoting weight regain [62]. Alterations in satiety hormones [63], delayed gastric emptying triggered by weight loss leading to delayed release of satiety hormones [64], and an increase in appetite following all the above-mentioned physiological changes might be factors contributing to the weight regain [65]. Furthermore, when comparing weight changes between pre-treatment BMI categories and treatment methods, participants in the underweight, normal, and overweight categories gained the most weight by switching to a higher BMI category across both treatment modalities. The rehabilitation group had a higher prevalence of this change in BMI. Finally, the majority of the participants who were initially obese maintained their status. Peles et al. [55] and Sweeney et al. [29] reported an increase in the number of patients classified as overweight or obese, and a decrease in the number of patients classified as normal weight nine months post admission to MMT, which supports our findings. Moreover, Montazerifar et al. [54] showed that the percentage of patients in MMT who were initially categorized as being overweight or obese remained as such after eight weeks of treatment. In another study, the percentage of adolescents in residential rehabilitation treatment centers who are at risk of being overweight (BMI between 85 and 95% for gender and age) increased from 7.1% to 14.7% after three months of the treatment [66]. The treatment for drug use disorders is lifesaving but is associated with the prevalence of weight gain. Identifying protective factors to decrease the negative clinical risk factors is warranted.

It is worth noting that the majority of the observed weight gain and BMI increases in our sample were not due to undernourished drug users losing weight. Evidence suggests that unhealthy weight gain in this population is an overlooked problem. Obesity and overweight are common among the general population, and are risk factors for several diseases such as hypertension, diabetes, and cardiovascular disorders [67]. Alarmingly, it has been reported that patients with MMT show weight increase that is similar to that of the general population over time [55]. After three years of MMT, the percentage of patients who were obese or morbidly obese was higher than the percentage of adults in the United States whose weight was categorized as such [29]. Furthermore, the increase in BMI among adolescents in rehabilitation centers was above the expected 10% for a gender- and age-matched population [66]. The trend of overweight and obesity prevalence among PWUD in treatment for recovery is alarming due to its potential negative health outcomes. There is evidence of an increased prevalence of type 2 diabetes, hypertension, and hyperlipidemia among PWUD undergoing MMT [29,68,69]. This reported increase exceeds the general population estimates. Prospective long-term studies examining hard outcomes in this population, on the other hand, are scarce. Longitudinal studies examining weight change patterns, characterizing the risk factors linked to weight gain, and considering the implications of this weight gain on health at different treatment intervals are warranted.

Participants with a higher pre-treatment BMI experienced less weight gain during treatment. Similar to our results, Peles et al. [55] reported that the BMI of patients categorized as initially high (BMI ≥ 27 Kg/m^2^) in MMT did not change significantly over the course of treatment. Furthermore, studies on normal populations found that thin people gain weight more easily than obese individuals [70]. Future research on the association of pre-treatment BMI among PWUD in recovery is needed to generalize the findings.

In our study, participants in rehabilitation had a more significant weight gain than people receiving OST. To our knowledge, no studies in the literature have compared the two types of treatments in terms of weight gain. A possible explanation is that OST is an unsupervised program where participants return home after receiving their dose of treatment, hence they might not be fully complying with abstinence from drugs, causing decreased food consumption and a lack of regular eating patterns [4]. Furthermore, our unpublished data on PWUD undergoing OST in Lebanon revealed that the majority of the participants experienced dysfunctional family relationships, criminal justice involvement, and insufficient income, all of which make eating healthy a challenge and exacerbate food insecurity. On the other hand, residential treatment centers are strictly supervised so that complete abstinence from drugs is secured. Participants follow a disciplined routine that includes three communal meals at fixed times and a structured physical activity program [13,37]. Considering this, one would assume that this would be ideal for promoting better health in patients, but binging on sugary and high-fat foods has been observed as a compensation for drug use and as a result of boredom from the strict environment [37,71]. Another explanation for this higher weight gain is the highly processed foods offered instead of healthy options due to the limited financial resources of these institutions [37,57]. Finally, weight gain is perceived by underweight participants as a healthy indicator that was targeted and well received in the early phases of the treatment [36,37]. Despite this significant difference in weight gain across treatment modalities, the type of treatment did not appear to be an independent predictor of weight gain among this population group in the multivariate analysis. This could be attributed to the small sample size in each group. Future studies are warranted to further investigate whether there is a need to tailor different intervention programs addressing all risk factors associated with weight gain, based on the type of treatment offered. Rehabilitation centers offer a stable environment for targeting health risk behaviors among a homogenous population with common features and support systems. On the other hand, OST could benefit from a more personalized approach to intervention, educating patients on coping mechanisms without direct supervision.

Weight increase was adversely correlated with the number of prior treatments tried as well as the length of those treatments. Our qualitative research with PWUD in rehabilitation centers revealed that participants, after several treatment attempts, become aware of the weight gain that they will face in the early phases of rehabilitation and of the difficulties in losing it at later stages. As a result, they become more conscious of their food binging [37]. Moreover, it is possible that the weight acquired during the previous treatment attempts made it harder specifically for obese and overweight participants to gain further weight [70,72]. These findings need to be further explored as they could inform weight management prevention efforts in this population. Furthermore, participants with previous exposure to weight gain may provide positive peer support to cope with cravings. Their prior knowledge of the obstacles faced in weight loss in later stages of the treatment is likely to ensure better coping strategies in the weight gain intervention among their peers. Furthermore, and in support to our results, studies show that weight and eating behavior changes differed by the stage and duration of recovery [1,13]. Regaining weight lost during active drug use was dominant in the early phases of treatment and exceeded the weight lost during substance abuse [13]. The urge to maintain body weight at its appropriate biological level or to the compromised neurobiological system, making food a compensation for the drug may be the cause of this increase in food consumption and weight [73]. The dysfunctional eating practices and cravings improve in the mid- and late- recovery phases, where the weight gained becomes a concern and a cause for anxiety and distress [1]. This finding should be confirmed by longitudinally measured weights in treatment centers and may suggest that any intervention among this population group should take the recovery stages into consideration.

Looking at other nutrition and lifestyle practices, our findings showed that nutrition knowledge, food addiction, physical activity level, and sleep quality were not associated with weight gain. Similarly, Sason et al. [16] showed that nutrition intervention programs among patients undergoing MMT led to increased nutrition knowledge without showing weight loss or changes in BMI. In contrast, Peles et al. [55] showed that participants undergoing MMT with a high BMI scored lower on knowledge about a healthy diet compared with those with a lower BMI. Furthermore, there is no conclusive evidence regarding the association between nutrition knowledge and healthy weight among the general population. Nutritional knowledge of obese individuals was comparable with that of healthy individuals, suggesting that there may be other factors accounting for the higher BMI [74]. While education and knowledge provide the skills to choose better food options, it has been proposed that knowledge alone, although necessary, is typically not enough to facilitate behavior change [75]. Addressing personal, behavioral, and environmental barriers to dietary behavior change is an important component, in addition to nutrition knowledge, in any future health promotion model among this group [76].

Food addiction was another factor that did not show an association with weight gain in our study. The literature shows conflicting results regarding the correlation of food addiction and weight gain among overweight and obese individuals in the general population [21,77,78]. According to Pursey et al. [79], 24.9% of obese and overweight individuals suffered from food addiction, compared to 11.1% in healthy weight persons. To our knowledge, there are no studies associating food addiction with weight gain among PWUD. Drug use and food addiction have similar neurobiological pathways regarding expressions of dopamine. Repeated exposures to high-sugar and high-fat foods reduce dopamine release, triggering the need for higher intakes and thus weight gain [79,80]. Although food addiction has been seen in people of all weight classes, it remains more common in obese people [81]. Therefore, obesity should not be used as a representation of food addiction, and longitudinal studies are needed to determine the temporal relation between food addiction and weight gain.

Furthermore, weight increase was not linked to physical exercise in our sample. The importance of physical activity as a behavioral strategy for preventing weight gain has been emphasized in the general population [82,83]. The most consistent finding in cross-sectional studies is that a high level of physical activity is linked to reduced weight gain [84,85,86]. On the other hand, randomized trials with physical exercise interventions show inconsistent results about this association [87]. Physical activity as a determinant of weight gain in PWUD patients undergoing treatment for recovery has not been addressed. One explanation for the lack of association seen in our sample could be due to the fact that it was not quantitatively measured but assessed using questionnaires depending on the participants’ recall. Another explanation could be the high energy intake among PWUD in treatment [37]. Physical activity in PWUD patients undergoing treatment has shown extensive benefits in terms of reducing withdrawal symptoms, anxiety, and depression [18,19]. Further longitudinal studies exploring the benefits of physical activity on weight gain reported in this population group are important. Moreover, changes in body composition, specifically increases in muscle mass, resulting from physical activity should be investigated. Rehabilitation centers are controlled environments for implementing behavioral changes and acceptance of healthy lifestyle intervention programs, including structured physical activity and adequate nutritional meals, as compared to OST centers.

Finally, there was no association between sleep and weight increase. Little attention has been paid to PWUD patients’ sleep while they are receiving recovery treatment. The dearth of studies focuses mainly on patients undergoing MMT who show poor quality and quantity of sleep arising from psychopathological problems or the methadone itself [17,88]. The published literature shows conflicting results supporting the association between sleep and weight gain in adults. Cross-sectional studies support the association between weight gain and poor sleep quality, whereas longitudinal studies have conflicting results, with some showing a diminishing association over time [89,90]. Short-sleeper individuals do not gradually gain weight linearly over time [91]. The longitudinal timeframe of the study would need to start at the beginning of the individual’s short sleep transition for short sleep to predict any physiological or behavioral change [92]. This could support our results because the sleep deprivation of PWUD begins during active drug use and continues throughout treatment. The weight gain observed could be attributed to the association with poor sleep quality at the beginning of the sleep deprivation and not at the time of the data collection. A possible mechanism attributed to this weight gain is that lack of sleep leads to hormonal imbalances with decreases in leptin and increases in ghrelin, leading to increases in food intake [93]. Sleep as a determinant of weight gain among PWUD in treatment centers has not been addressed. Further studies are warranted to confirm its association with weight gain over time.

### 4.1. Strengths and Limitations

This study is a pioneer in exploring the patterns of weight change and identifying the determinants of weight gain reported among PWUD patients undergoing treatment for recovery in the MENA region, specifically in Lebanon. Moreover, it fills a gap in the literature by identifying weight change patterns across BMI categories and the quality of sleep specifically in rehabilitation centers. Drug use has progressed tremendously in Lebanon after the civil war, due to a more permissive sociocultural context. Furthermore, a growing demand for treatment centers in the country has been observed recently, owing primarily to the suspension of all judicial decisions against persons seeking treatment [40]. For these reasons, addressing this population group is critical in order to develop health intervention programs addressing risk factors associated with the observed increase in weight gain. This study thus provides useful information in this regard. Additionally, this study fills a gap in the literature comparing the two treatment modalities. This may play an important role in customizing intervention programs offered based on the type of treatment.

On the other hand, this study has some limitations. First, one important limitation is that the weight gain seen in our sample was reported and not measured. This study is a cross-sectional one, and the participants were met for the first time during the data collection. Second, the weight of the participants before drug use is not available to compare to the weight during drug use and in treatment, as most participants had been using drugs for a very long period of time and did not remember their previous weight. Third, although the assessment tools used, such as the YFAS [47], PSQI [48], and IPAQ [49], were validated, further validation among the population of PWUD is warranted, and the information provided is not measured, rather it was on a recall basis. Fourth, poor lifestyle factors, including alcohol and cigarette smoking, were not adequately addressed in this study and may be contributing factors to weight gain. Fifth, the dietary intake was measured once by 24 h recall. This might not be representative of the participants’ usual intake and is subject to recall and social desirability bias [94]. Sixth, female participants were not represented in the sample due to the limited number of female residential treatment centers and the fear of stigmatization among the OST group. Finally, we used the stepwise method in the multivariate regression analysis. This could be debated by the fact that it is recommended in exploratory and predictive research, where subject matter knowledge is limited, as it was in our study [95]. The interpretation of results should only be preliminary and should not assign meaningfulness to the order of variable entry and selection [96].

### 4.2. Future Studies

Comparative studies between Lebanon and elsewhere should be conducted to assess whether our findings are shared features among PWUD in treatment for recovery and are not specific only to the Lebanese population. Furthermore, longitudinal studies investigating the consequences of weight gain on disease risk factors and hard outcomes among this population group at different treatment intervals are warranted. In addition to this, future studies investigating the determinants of weight gain in each treatment modality separately are warranted. Moreover, validation of the tools used by PWUD is important for future research among this population group. Furthermore, retrospective studies examining weight change from the onset of poor sleep during active drug use and across treatment to confirm association are being conducted. Finally, studies aiming at the development of health interventions targeting the significant weight gain during treatment for drug use disorders are of great importance.

## 5. Conclusions

Treatment from drug use is lifesaving, but it is associated with meaningful weight gain that might lead to health risk factors. This weight gain was higher in the rehabilitation group and among people in the underweight, normal, and overweight BMI categories. PWUD in treatment for recovery are a vulnerable group with poorly served needs. Treatment centers could benefit from further improvement to better serve their patients’ needs, particularly with regard to nutrition and lifestyle parameters. Developing health promotion programs with the goal of enhancing the therapeutic process, reducing health risk factors, and preventing relapse.

## Figures and Tables

**Table 1:** Demographics characteristics and drug use history of the participants (*n* = 172).

	OST (*n* = 94)		Rehabilitation (*n* = 78)		*p*-Value	Total (*n* = 172)	
	Mean	SD	Mean	SD		Mean	SD
Age (years)	33.7	8.3	30.5	8.3	0.007 **	33.0	8.6
	N	%	N	%		N	%
Educational level							
Illiterate	8.0	8.5	2.0	2.6	0.446	10.0	5.8
Elementary/intermediate	35.0	37.2	31.0	39.7		66.0	38.4
Secondary	26.0	27.7	22.0	28.2		48.0	27.9
University	25.0	26.6	23.0	29.5		48.0	27.9
Occupation							
Unemployed/Retired	38.0	40.4	38.0	48.7	0.07	76.0	44.2
Employed	27.0	28.7	15.0	19.2		42.0	24.4
Self-employed	29.0	30.9	20.0	25.6		49.0	28.5
Student	0.0	0.0	4.0	5.1		4.0	2.3
Other	0.0	0.0	1.0	1.3		1.0	0.6
Marital status							
Single	63.0	67.0	61.0	78.2	0.221	124.0	72.1
Married	23.0	24.5	11.0	14.1		34.0	19.8
Divorced/separated	8.0	8.5	6.0	7.7		14.0	8.1
Current housing							
Residence	94.0	100.0	24.0	30.8	<0.001	118.0	68.6
Rehabilitation	0.0	0.0	54.0	69.2		54.0	31.4
People with whom the participant stays: pre-treatment (rehabilitation) and currently (OST)							
Alone	7.0	7.4	4.0	5.1	<0.001	11.0	6.4
Spouse/partner	26.0	27.7	2.0	2.6		28.0	16.3
Parents	59.0	62.8	13.0	16.7		72.0	41.9
Relative/colleagues	2.0	2.1	56.0	71.8		58.0	33.7
No response	0.0	0.0	3.0	3.8		3.0	1.7
Medications used							
Antidepressants	16.0	17.0	23.0	29.5	0.067	39.0	22.7
Antipsychotic	30.0	31.9	31.0	39.7	0.337	61.0	35.5
Epilepsy-bipolar	11.0	11.7	24.0	30.8	0.002	35.0	20.3
Previous treatment							
None	35.0	37.2	41.0	52.6	0.002	76.0	44.2
OST	6.0	6.4	4.0	5.1		10.0	5.8
Rehabilitation	22.0	23.4	27.0	34.6		49.0	28.5
Rehabilitation and OST	10.0	10.6	3.0	3.8		13.0	7.6
Hospital detoxification	17.0	18.1	2.0	2.6		19.0	11.0
Hospital detoxification and rehabilitation	4.0	4.3	0.0	0.0		4.0	2.3
No response	0.0	0.0	1.0	1.3		1.0	0.6
Other addiction							
None	87.0	92.6	46.0	59.0	<0.001	133.0	77.3
Alcohol	1.0	1.1	25.0	32.1		26.0	15.1
Other	6.0	6.4	7.0	9.0		13.0	7.6
	Mean	SD	Mean	SD		Mean	SD
Duration of drug use (years)	11.5	7.2	11.4	7.5	0.782	12.8	7.7
Duration of drug injection (years) (among those who reported drug injection)	7.4	6.3	8.6	5.7	0.352	7.7	6.2
Number of previous treatment attempts	3.7	4.9	2.0	2.4	0.052	3.1	4.4
Treatment duration (months)	31.6	25.6	5.5	5.5	<0.001 **	24.9	27.9

OST, Opioid substitution treatment. **, *p* < 0.05 using Mann–Whitney U test, *p* < 0.05 using chi-Square.

**Table 2:** Anthropometrics and lifestyle practices of the participants (*n* = 172).

	OST (*n* = 94)		Rehabilitation (*n* = 78)		*p*-Value	Total (*n* = 172)	
	Mean	SD	Mean	SD		Mean	SD
Pre-treatment BMI (Kg/m^2^)	25.9	4.7	24.1	5.0	0.016 *	25.9	5.2
During-treatment BMI (Kg/m^2^)	26.6	5.3	27.6	4.6	0.209	27.4	5.5
Energy (Kcal)	2781.8	1485.4	2480.4	892.9	0.548	2641.9	1251.5
Energy (Kcal/Kg)	35.4	21.1	30.0	12.0	0.297	32.9	16.6
	N	%	N	%		N	%
Weight change							
Weight loss	31.0	33.0	7.0	9.0	<0.001	38.0	22.1
No change	12.0	12.8	10.0	12.8		22.0	12.8
Weight gain	51.0	54.3	61.0	78.2		112.0	65.1
Sleep quality index							
Good sleep quality	28.0	30.1	14.0	17.9	0.076	42.0	24.6
Poor sleep quality	65.0	69.9	64.0	82.1		129.0	75.4
Physical activity level							
Low activity level	68.0	72.3	19.0	24.4	<0.001	87.0	50.6
Moderate activity level	19.0	20.2	24.0	30.8		43.0	25.0
High activity level	7.0	7.4	35.0	44.9		42.0	24.4
Food addiction							
No diagnosis met	50.0	53.2	36.0	48.0	0.538	86.0	50.9
Diagnosis met	44.0	46.8	39.0	52.0		83.0	49.1
Nutrition knowledge							
Poor nutrition knowledge	69.0	73.4	49.0	62.8	0.142	118.0	68.6
Good nutrition knowledge	25.0	26.6	29.0	37.2		54.0	31.4

OST, Opioid substitution treatment. *, *p* < 0.05 using independent samples *t*-test.

**Table 3:** Total weight change (kg), and weight change per pre-treatment BMI categories and treatment method.

	OST (*n* = 94)			Rehabilitation (*n* = 78)			Total (*n* = 172)		
	N	Mean	SD	N	Mean	SD	N	Mean	SD
Underweight	1.0	17.0		9.0	24.5	12.3	10.0	23.8	11.9
Normal	40.0	1.6	5.5	40.0	12.4	10.4	80.0	7.0	9.9
Overweight	40.0	4.8	12.1	17.0	4.2	8.9	57.0	4.6	11.2
Obese	13.0	−6.1	17.3	12.0	3.4	10.5	25.0	−1.56	15.0
Total	94.0	2.0	11.3	78.0	10.6	12.0	172.0	5.9	12.4

BMI, Body mass index; OST, Opioid substitution treatment.

**Table 4:** Bivariate analysis of demographics, drug use, treatment methods, anthropometrics, nutrition and lifestyle practices with weigh gain (*n* = 172).

	No Weight Gain (*n* = 60)		Weight Gain (*n* = 112)		*p*-Value
	Mean	SD	Mean	SD	
Age in years	33.2	8.1	31.8	8.5	0.309
Duration of drug use (years)	12.5	8.3	10.9	6.6	0.197
Number of previous treatment attempts	2.6	5.0	0.9	1.8	0.022 *
Duration of current treatment (months)	25.5	27.4	16.6	20.1	0.030 *
Energy (Kcal/Kg)	36.0	20.3	31.3	16.0	0.108
Protein (g/Kg)	1.1	0.6	1.0	0.6	0.220
Fiber (g)	21.9	13.1	22.4	11.3	0.772
	% From total calories	SD	% From total calories	SD	
Carbohydrate	48.6	9.7	49.6	10.4	0.578
Added sugar	2.8	4.0	2.5	3.3	0.673
Fat	38.7	9.4	38.1	10.1	0.693
	N	%	N	%	
Educational level					
Illiterate	2.0	3.3	8.0	7.1	0.735
Elementary/intermediate	23.0	38.3	43.0	38.4	
Secondary	16.0	26.7	32.0	28.6	
University	19.0	31.7	29.0	25.9	
Type of treatment					
OST	43.0	71.7	51.0	45.5	0.001 *
Rehabilitation	17.0	28.3	61.0	54.5	
Current use of antidepressants	11.0	18.3	28.0	25.0	0.347
Current use of antipsychotics	16.0	26.7	45.0	40.2	0.095
Current use of epilepsy/bipolar medications	10.0	16.7	25.0	22.3	0.432
Current use of any medications	24.0	40.0	58.0	51.8	0.152
Pre-treatment BMI (kg/m^2^)					
Underweight (%)	0.0	0.0	10.0	8.9	0.003 *
Normal weight (%)	22.0	36.7	58.0	51.8	
Overweight (%)	24.0	40.0	33.0	29.5	
Obesity (%)	14.0	23.3	11.0	9.8	
Food addiction					
No diagnosis met	30.0	52.6	56.0	50.0	0.871
Diagnosis met	27.0	47.4	56.0	50.0	
Nutrition knowledge					
Poor knowledge	40.0	66.7	78.0	69.6	0.732
Good knowledge	20.0	33.3	34.0	30.4	
Sleep quality index					
Good sleep quality	18.0	30.0	24.0	21.6	0.265
Poor sleep quality	42.0	70.0	87.0	78.4	
Physical activity level					
Low	33.0	55.0	54.0	48.2	0.408
Moderate	16.0	26.7	27.0	24.1	
High	11.0	18.3	31.0	27.7	

OST, opioid substitution treatment; BMI, body mass index. * *p* < 0.05.

**Table 5:** Multivariate logistic regression of determinants of weight gain.

	Weight Gain (Reference: No)			
	OR	95% CI		*p*-Value
		Lower	Upper	
Number of previous treatments	0.86	0.74	0.99	0.043
Duration of current treatment (months)	0.98	0.96	0.99	0.015
Pre-treatment BMI (Kg/m^2^)	0.86	0.79	0.95	0.003

Variables included in the model were: number of previous treatment attempts; duration of current treatment (months); energy (kcal/kg); type of treatment (reference: OST); current use of any medication (reference: no); pre-treatment BMI (kg/m^2^); food addiction (reference: no diagnosis met); nutrition knowledge (reference: good knowledge); sleep quality (reference: good sleep quality); physical activity (reference: high). OR: odds ratio; CI: confidence interval; OST, opioid substitution treatment; BMI, body mass index.

## Data Availability

Data is unavailable due to the privacy of this vulnerable population.
